# Influence of Angptl1 on osteoclast formation and osteoblastic phenotype in mouse cells

**DOI:** 10.1186/s12891-021-04278-6

**Published:** 2021-04-28

**Authors:** Masayoshi Ishida, Naoyuki Kawao, Yuya Mizukami, Yoshimasa Takafuji, Hiroshi Kaji

**Affiliations:** Department of Physiology and Regenerative Medicine, Kindai University Faculty of Medicine, 377-2 Ohnohigashi, Osakasayama, Osaka, 589-8511 Japan

**Keywords:** Angptl1, Osteoblast, Osteoclast, Adipocyte, Bone

## Abstract

**Background:**

Osteoblasts and osteoclasts play important roles during the bone remodeling in the physiological and pathophysiological states. Although angiopoietin family Angiopoietin like proteins (Angptls), including Angptl1, have been reported to be involved in inflammation, lipid metabolism and angiogenesis, the roles of Angptl1 in bone have not been reported so far.

**Methods:**

We examined the effects of Angptl1 on the osteoblast and osteoclast phenotypes using mouse cells.

**Results:**

Angptl1 significantly inhibited the osteoclast formation and mRNA levels of tartrate-resistant acid phosphatase and cathepsin K enhanced by receptor activator of nuclear factor κB ligand in RAW 264.7 and mouse bone marrow cells. Moreover, Angptl1 overexpression significantly enhanced Osterix mRNA levels, alkaline phosphatase activity and mineralization induced by bone morphogenetic protein-2 in ST2 cells, although it did not affect the expression of osteogenic genes in MC3T3-E1 and mouse osteoblasts. On the other hand, Angptl1 overexpression significantly reduced the mRNA levels of peroxisome proliferator-activated receptor γ and adipocyte protein-2 as well as the lipid droplet formation induced by adipogenic medium in 3T3-L1 cells.

**Conclusions:**

The present study first indicated that Angptl1 suppresses and enhances osteoclast formation and osteoblastic differentiation in mouse cells, respectively, although it inhibits adipogenic differentiation of 3T3-L1 cells. These data suggest the possibility that Angptl1 might be physiologically related to bone remodeling.

## Background

Bone is a living organ that undergoes remodeling throughout life. Bone remodeling is accelerated by the bone resorption by osteoclasts and subsequent bone formation by osteoblasts under the influences of growth factors, cytokines and/or chemokines, such as bone morphogenetic protein (BMP)-2 and stromal derived factor-1 [1, 2]. Osteoclasts and osteoblasts are originated from granulocyte/macrophage-lineage hematopoietic stem cells and mesenchymal stem cells, respectively. In homeostatic balance, bone resorption and formation are balanced so that old bone is continuously replaced by new tissue to accommodate mechanical loading and strain [3]. The remodeling cycle consists of three consecutive phases: absorption, inversion, and formation.

Angiopoietin like proteins (Angptls) contain eight secreted glycoproteins, namely Angptl1 to Angptl8, showing structural similarity to members of angiopoietin family proteins [4]. All Angptls have an amino-terminal coiled-coil domain, a linker region, and a carboxy-terminal fibrinogen-like domain, and bind to the Tie2 receptor similarly to angiopoietin except for Angptl8 (without the carboxy-terminal fibrinogen-like domain). Angiopoietin and Angptls show high similarity among them [5]. Angptls are widely expressed in various tissues, including liver, cardiovascular and hematopoietic system, and play important roles in inflammation, lipid metabolism and angiogenesis [6].

Angiopoietin like protein-1 (Angptl1) with 491 amino acids has firstly been an isolated cDNA as encoding one of the novel angiopoietin like protein family members from human adult heart cDNA. Angptl1, also known as angioarrestin, was the first discovered member of the Angptl family, since it was identified as an anti-angiogenic factor that inhibits cell proliferation, migration and adhesion of endothelial cells [5, 7]. Angptl1 inhibits vascular endothelial growth factor and basic fibroblast growth factor-induced proliferation of the endothelial cells and exerts the anti-apoptotic action through an induction of phosphorylation of extracellular-signal-regulated kinase 1/2 (ERK1/2) and protein kinase Bα in hepatocellular carcinoma [8]. Moreover, Angptl1 and Angptl2 have been reported to be involved in metabolic syndrome in human and rodents [9]. Lin et al. reported that Angptl1/2 enhances the formation of hematopoietic stem and progenitor cells through the interaction with notch receptor signaling in zebrafish [10]. These findings suggest that Angptl1 is related to wide variety of pathophysiological process in various diseases.

Although angiopoietin and Angptls are known as angiogenic growth factors, the studies of Angptl1, Angptl2 and Angptl1/Angptl2-double knockout zebrafish and mice clearly demonstrated that Angptl2 and Angptl1 have angiogenic and anti-angiogenic function in endothelial cells, respectively, although only double knockout zebrafish showed the defects of endothelial development from embryos [11]. Although vessel formation and blood supply are important for the maintenance and activation of bone remodeling, it has not been reported about the roles of Angptl1 in bone metabolism elsewhere. We therefore investigated the effects of Angptl1 on osteoclast formation, osteoblastic differentiation and osteoblast phenotypes using mouse cell-lines and primary cultured cells.

## Materials and methods

### Ethics

All animal experiments were performed according to the guidelines of the National Institutes of Health and the institutional rules for the use and care of laboratory animals at Kindai University. The procedures of animal experiments were approved by the Experimental Animal Welfare Committee of Kindai University (KAME-2020-011). The study was carried out in compliance with the ARRIVE guidelines.

### Materials

Recombinant Angptl1 was obtained from Cloud-Clone Corp (Katy, TX, USA). Receptor activator of nuclear factor κB ligand (RANKL) and macrophage colony stimulating factor (M-CSF) were purchased by Wako Pure Chemicals (Osaka, Japan). BMP-2 was obtained from Pfizer (Groton, CT, USA). Anti-phosphorylated p65 (Cat. No., 3033), anti-p65 (Cat. No., 8242) and anti-glyceraldehyde-3-phosphate dehydrogenase (GAPDH, Cat. No. 5174) antibodies were purchased from Cell Signaling Technology (Danvers, MA, USA).

### Cell culture

Mouse osteoblastic MC3T3-E1 cells (provided by Dr. Kodama, Ohu Dental Collage, Koriyama, Japan) were cultured in Minimum Essential Medium Alpha (αMEM) with 10% fetal bovine serum (FBS) and 100 units/ml penicillin-100 μg/ml streptomycin. ST2 cells (RIKEN, Tsukuba, Japan), a mouse mesenchymal cell line, were maintained in RPMI1640 with 10% FBS and 100 units/ml penicillin-100 μg/ml streptomycin. Mouse monocytic RAW 264.7 cells (ATCC, Manassas, VA, USA) and mouse preadipocytic 3T3-L1 cells (ATCC) were cultured in Dulbecco’s Modified Eagle’s Medium (DMEM) with high glucose, 10% FBS and 100 units/ml penicillin-100 μg/ml streptomycin.

### DNA construction

For DNA construction, the coding region of murine Angptl1 was amplified by PCR with KAPA HiFi DNA Polymerase enzyme (KAPA Biosystems, Wilmington, MA, USA), using cDNA from mouse neonatal liver as a template and primers (Forward: ATAGAGGCTAGCATGAAGGCTTTTGTTTGGAC, Reverse: ATCAACTCGAGTCAGTCAATAGGCTTGATCA), and then the resultant fragment was inserted into pcDNA3.1 (+) vector (Invitrogen, Thermo Fishers Scientific, Waltham, MA, USA) at the NheI/XhoI sites, and the DNA sequence was verified.

### Transfection

For transient transfection, the expression vector was transfected into cells using jetPRIME reagent (Polyplus-transfection SA, Illkirch, France) according to the manufacture’s protocol. Six hours later, the cells were supplied with fresh αMEM, RPMI1640 or DMEM with high glucose containing 10% FBS. Forty-eight hours later, the transiently transfected cells were used for experiments.

For stable transfection, empty vector or pcDNA3.1-Angptl1 vector was transfected into ST2 cells using jetPRIME reagent, as described above. The cells were then subjected to the RPMI1640 supplemented with 10% FBS and 300 μg/ml G418 after 48 h transfection. The several colonies were obtained and the expression levels of Angptl1 were determined by quantitative reverse transcriptase-polymerase chain reaction (qRT-PCR) method.

### Osteoclast formation

RAW 264.7 cells were seeded onto 96-well plate at 1000 cells/well. Empty vector- or Angptl1-transfected RAW 264.7 cells were cultured with αMEM containing 10% FBS and 50 ng/ml of RANKL to induce osteoclast differentiation for 5 days. For osteoclast formation in mouse bone marrow cells, cells were collected from tibias of 6 to 8-week-old male C57BL/6 J mice. Osteoclast formation was induced in mouse bone marrow cells, as described previously [12]. Mouse bone marrow cells were cultured in αMEM with 10% FBS and 50 ng/ml M-CSF for 3 days. Then, osteoclasts were formed in αMEM with 10% FBS, 50 ng/ml M-CSF and 50 ng/ml RANKL for a further 4 days. The cells were fixed with 4% formaldehyde, and tartrate-resistant acid phosphatase (TRAP) staining were performed with TRAP Stain kit (WAKO Pure Chemicals) according to manufacturer’s protocol. TRAP-positive multinuclear cells (MNCs) with three or more nuclei were counted as osteoclasts.

### qRT-PCR

Total RNA was extracted from cells using TriSure reagent (Nippon Genetics, Tokyo, Japan) in accordance with the manufacturer’s instructions and real-time PCR was performed as described [13]. Each PCR primer set is shown in Table 1. Expression levels of mRNA were normalized to the relative amount of a housekeeping gene, GAPDH or β-actin. Each experiment was performed in duplicate.

**Table 1:** Primers used for quantitative RT-PCR experiments

Gene		Sequence
Angptl1	Forward	CGACACAGTCCTAACAGCCACCAG
	Reverse	TGACAGTCTTTGAATGGTCCTTC
TRAP	Forward	CAGCTGTCCTGGCTCAAAA
	Reverse	ACATAGCCCACACCGTTCTC
Ctsk	Forward	GAGGGCCAACTCAAGAAGAA
	Reverse	GCCGTGGCGTTATACATACA
NFATc1	Forward	CAAGTCTCACCACAGGGCTCACTA
	Reverse	GCGTGAGAGGTTCATTCTCCAAGT
MMP-9	Forward	GGACCCGAAGCGGACATTG
	Reverse	CGTCGTCGAAATGGGCATCT
DC-STAMP	Forward	CTAGCTGGCTGGACTTCATCC
	Reverse	TCATGCTGTCTAGGAGACCTC
Runx2	Forward	AAATGCCTCCGCTGTTATGAA
	Reverse	GCTCCGGCCCACAAATCT
Osterix	Forward	AGCGACCACTTGAGCAAACAT
	Reverse	GCGGCTGATTGGCTTCTTCT
ALP	Forward	ATCTTTGGTCTGGCTCCCATG
	Reverse	TTTCCCGTTCACCGTCCAC
Osteocalcin	Forward	CCTGAGTCTGACAAAGCCTTCA
	Reverse	GCCGGAGTCTGTTCACTACCTT
PPARγ	Forward	CCCAATGGTTGCTGATTACAAA
	Reverse	AATAATAAGGTGGAGATGCAGGTTCT
aP2	Forward	CTTCAAACTGGGCGTGGAA
	Reverse	CCATCTAGGGTTATGATGCTCTTCA
GAPDH	Forward	ACGGCAAATTCAACGGCAC
	Reverse	CTCCACGACATACTCAGCAC
β-actin	Forward	TACCACAGGCATTGTGATGG
	Reverse	TTTGATGTCACGCACGATTT

Angptl1, Angiopoietin like 1; TRAP, Tartrate-resistant acid phosphatase; Ctsk, cathepsin K; NFATc1, nuclear factor of activated T-cells, cytoplasmic 1; MMP-9, matrix metalloproteinase-9; DC-STAMP, dendrocyte expressed seven transmembrane protein; Runx2, Runt related transcription factor-2; ALP, alkaline phosphatase; PPARγ, peroxisome proliferator-activated receptor γ; aP2, adipocyte protein-2; GAPDH, glyceraldehyde-3-phosphate dehydrogenase

### Western blot

Western blot analysis was performed, as described previously [12]. Cells were lysed with radio-immunoprecipitation assay buffer containing protease inhibitor cocktails (Millipore, Bedford, MA, USA) and phosphatase inhibitor cocktails (Wako Pure Chemicals). The lysate was sonicated and centrifuged at 10000 rpm for 10 min. The supernatant was collected and equal amount of protein aliquots were separated by electrophoresis on 4–20% FastGene gradient gels (Nippon Genetics, Tokyo, Japan). The proteins were transferred onto a polyvinyl difluoride membrane (Millipore). The membrane was blocked with 3% skim milk and subsequently incubated with anti-phosphorylated-p65, anti-p65 or anti-GAPDH antibodies followed by horse radish peroxidase-labeled anti-rabbit IgG (Cell Signaling Technology). The bands were visualized using ImmunoStar Zeta (Wako Pure Chemicals) and captured with Amersham Imager 680 (GE Healthcare, Tokyo, Japan).

### Alkaline phosphatase (ALP) activity

Analysis of ALP activity in ST2 cells was performed, as described previously [14]. ST2 cells transiently expressing empty vector or Angptl1 were grown in 24-well plate. The confluent cells were stimulated with 200 ng/ml BMP-2 for 72 h. Then, the cells were lysed in distilled water and sonicated for 30 s on ice. After the lysates were centrifuged, ALP activity in the supernatant were analyzed using Lab assay ALP kit (Wako Pure Chemicals). The absorbance was measured at 405 nm by the Multiskan Go microplate spectrophotometer (Thermo Fishers Scientific). The protein levels in the supernatant were determined by Protein Assay BCA Kit (Wako Pure Chemicals). ALP activity was normalized to the protein levels and expressed as [units/total protein (μg)].

### Mineralization

To test the effect of Angptl1 on mineralization, stably empty vector- or Angptl1-overexpressed ST2 cells were grown in RPMI/10%FBS-penicillin/streptomycin containing 300 μg/ml G418 and 200 ng/ml BMP-2 for 1 week to induce osteoblasts, and then changed the mineralization media supplemented with 300 μg/ml G418 for additional 10 days. To detect mineralization level, the cells were fixed with 4% formaldehyde and stained with alizarin red. For the quantitation of mineralization, the resultant cells were destained with cetylpyridinium chloride and the absorbance at 550 nm were measured by the Multiskan Go microplate spectrophotometer (Thermo Fisher Scientific).

### Mouse primary osteoblasts

Primary osteoblasts were collected from the calvaria of new born C57BL/6 J mice, as described previously [15]. After mice were euthanized with excess isoflurane, calvaria was removed and cut into small pieces. The small pieces of calvaria were incubated four times with αMEM containing 1 mg/ml collagenase and 0.25% trypsin for 20 min at 37 °C. Cells from second, third and fourth digestions were collected and cultured in αMEM with 10% FBS and 100 units/ml penicillin-100 μg/ml streptomycin.

### Adipogenic differentiation

Confluent grown 3T3-L1 cells were transiently transfected with empty vector- or Angptl1 before the induction of adipogenic differentiation. Then, the medium was changed into high glucose DMEM-10%FBS supplemented with 10 μg/ml insulin, 500 μM 3-isobutyl-1-methyl-xanthine, and 1 nM dexamethasone for 2 days and then changed high glucose DMEM-10%FBS medium for additional 4 days as previously described [16]. The differentiated 3T3-L1 cells were fixed with 4% formaldehyde in phosphate-buffered saline for 30 min and stained with Oil red O solution according to the standard protocols. The stained cells were photographed under a microscope (BZ-700; Keyence, Osaka, Japan).

### Statistical analysis

Data are presented as mean ± SEM. Comparisons between two groups were made with the Mann-Whitney U test. For parametric multiple comparison, one-way or two-way analysis of variance followed by the Dunnett test or Tukey-Kramer test was employed by GraphPad PRISM 7.00 software. Significance was defined as *P* < 0.05. Each experiment was performed independently three times.

## Results

### Effects of Angptl1 on osteoclast formation

**Fig. 1:**
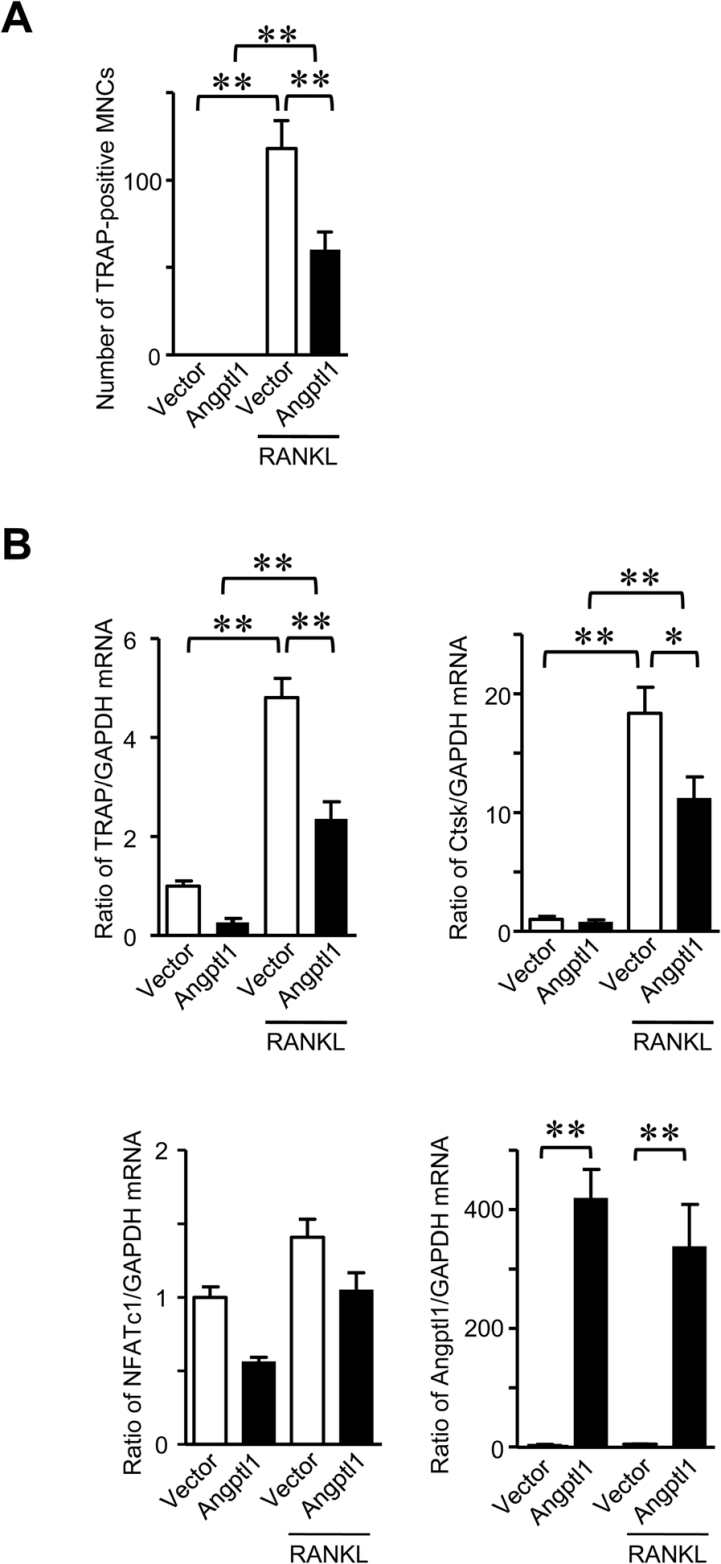
Effects of Angptl1 on osteoclast formation in RAW 264.7 cells. **a** Transiently empty vector- or Angptl1-transfected RAW 264.7 cells were cultured with or without 50 ng/mL RANKL for 5 days. Then, the number of TRAP-positive MNCs was counted. Data represent mean ± SEM (*n* = 5 in each group). **p < 0.01 by Tukey-Kramer test. **b** Total RNA was extracted from transiently empty vector- or Angptl1-transfected RAW 264.7 cells cultured with or without 50 ng/mL RANKL for 5 days. Then, qRT-PCR analysis for TRAP, cathepsin K (Ctsk), NFATc1, Angptl1 or GAPDH was performed. Data are expressed relative to GAPDH mRNA value. Data represent mean ± SEM (*n* = 6 in each group). *p < 0.05, **p < 0.01 by Tukey-Kramer test

**Fig. 2:**
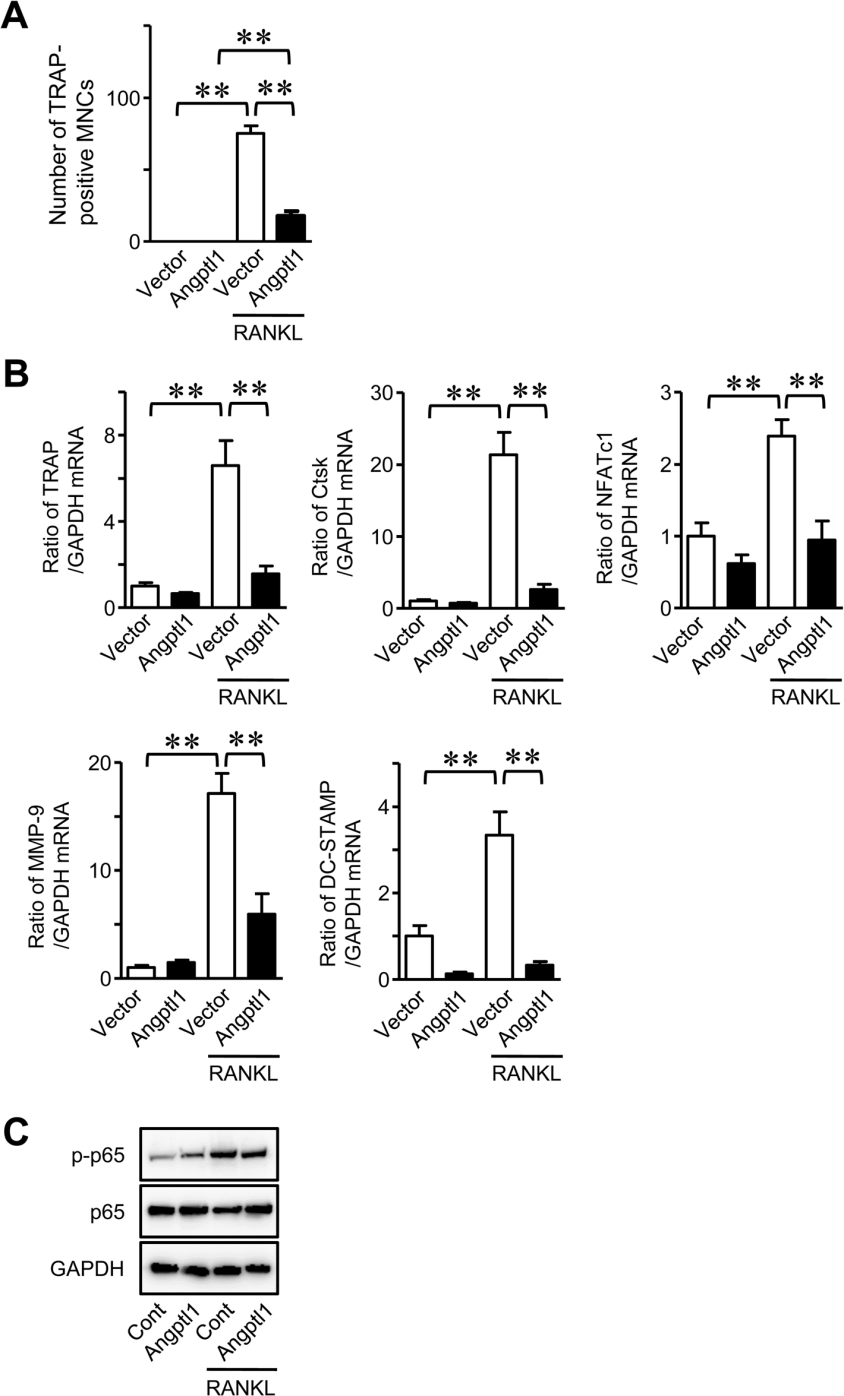
Effects of Angptl1 on osteoclast formation in mouse bone marrow cells. **a** Mouse bone marrow cells were pre-cultured with 50 ng/ml of M-CSF for 3 days, and further cultured with 50 ng/ml of RANKL and M-CSF in the presence or absence of recombinant Angptl1 (100 ng/ml) for additional 4 days. The number of TRAP-positive MNCs was counted. Data represent mean ± SEM (*n* = 6 in each group). **p < 0.01 by Tukey-Kramer test. **b** Mouse bone marrow cells were pre-cultured with 50 ng/ml of M-CSF for 3 days, and further cultured with 50 ng/ml of RANKL and M-CSF in the presence or absence of recombinant Angptl1 (100 ng/ml) for additional 4 days. Then, total RNA was extracted for qPCR analysis of TRAP, cathepsin K (Ctsk), NFATc1, MMP-9, DC-STAMP or GAPDH. Data are expressed relative to GAPDH mRNA value. Data represent mean ± SEM (n = 6 in each group). Two independent experiments were performed. **c** Mouse bone marrow cells were pre-cultured with 50 ng/ml of M-CSF for 3 days, and then cultured with or without recombinant Angptl1 (100 ng/ml) for 1 h in the presence of 50 ng/ml of RANKL and M-CSF. Total proteins were extracted from the cells and Western blotting analyses for phosphorylated-p65 (p-p65), p65 and GAPDH were performed. The images represent experiments performed independently 3 times. Cont; Control

We first examined the effects of Angptl1 on the osteoclast formation. Transient overexpression of Angptl1 significantly decreased the number of TRAP-positive MNCs enhanced by RANKL in mouse monocytic RAW 264.7 cells, compared with empty vector-transfected cells (Fig. 1a). Moreover, Angptl1 overexpression significantly downregulated the mRNA levels of osteoclastic markers, such as TRAP and cathepsin K (Ctsk), enhanced by RANKL, in RAW 264.7 cells, although it seemed to suppress nuclear factor of activated T-cells, cytoplasmic 1 (NFATc1) mRNA levels enhanced by RANKL without significant difference (Fig. 1b). As shown in Fig. 2, Angptl1 significantly suppressed number of TRAP-positive MNCs as well as the mRNA levels of TRAP, Ctsk, NFATc1, matrix metalloproteinase (MMP)-9 and dendrocyte expressed seven transmembrane protein (DC-STAMP) enhanced by RANKL in mouse bone marrow cells. It did not affect the phosphorylation of p65 enhanced by RANKL in these cells.

### Effects of Angptl1 on osteoblasts

**Fig. 3:**
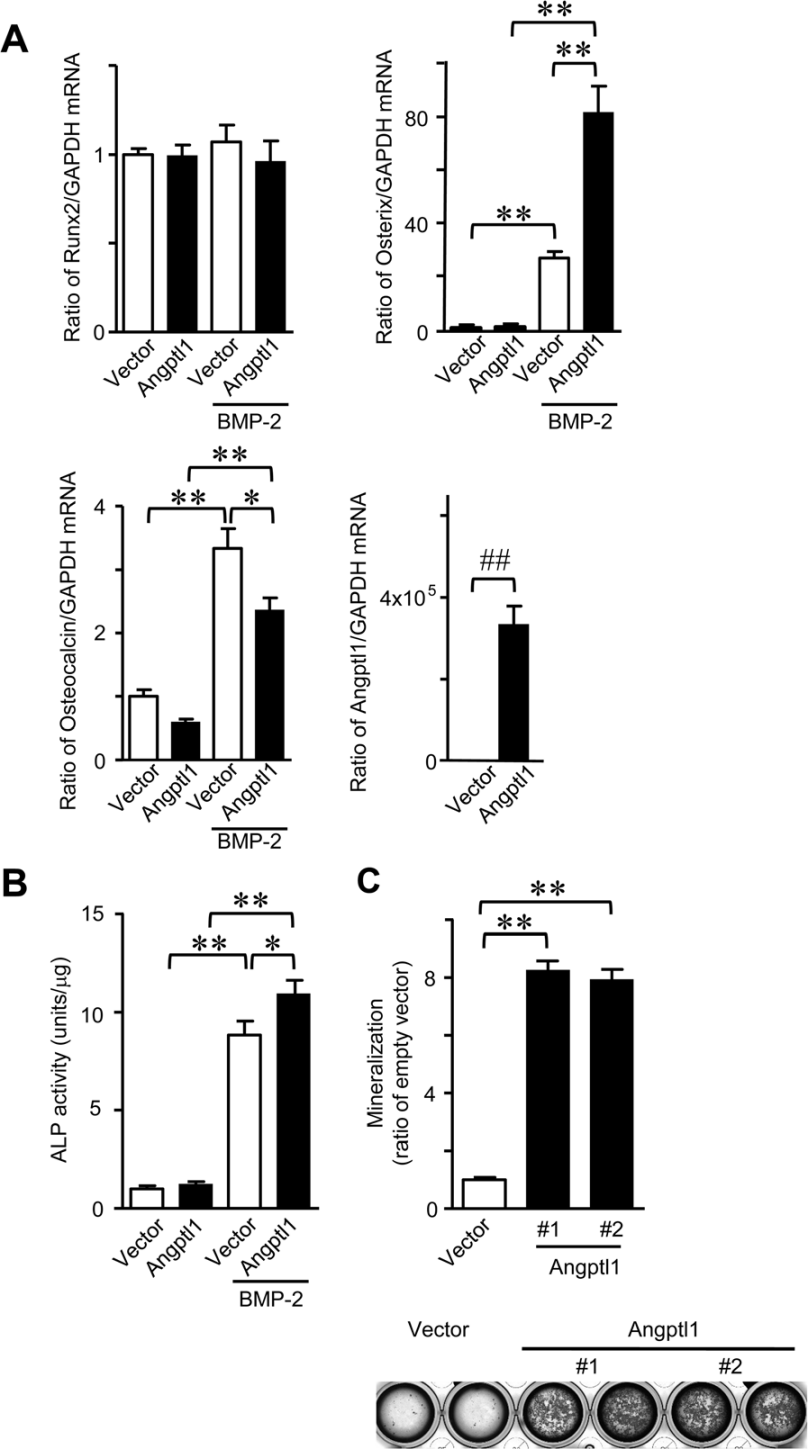
Effects of Angptl1 on osteoblastic differentiation. **a** Total RNA was extracted from transiently empty vector- or Angptl1-transfected ST2 cells cultured with vehicle or BMP-2 (200 ng/ml) for 72 h. Then, qRT-PCR for Runx2, Osterix, osteocalcin, Angptl1 or GAPDH was performed. Data are expressed relative to GAPDH mRNA value. Data represent mean ± SEM (*n* = 6 in each group). *p < 0.05, **p < 0.01 by Tukey-Kramer test. ^##^*p* < 0.01 by Mann-Whitney U test. **b** Cell lysates were extracted from transiently empty vector- or Angptl1-transfected ST2 cells cultured with vehicle or BMP-2 (200 ng/ml) for 72 h. Then, ALP activity was measured as described in Methods. Data represent mean ± SEM (*n* = 6 in each group). *p < 0.05, **p < 0.01 by Tukey-Kramer test. **c** Stably- empty vector- or Angptl1 (#1, #2)-transfected ST2 cells were treated with BMP-2 (200 ng/ml) and G418 (300 μg/ml) for 1 week, then cultured with the mineralization medium (10 mM β-glycerophosphate and 50 μg/ml ascorbic acid) and G418 (300 μg/ml) for additional 10 days in the absence of BMP-2. The quantitation of mineralization was performed at the absorbance at 550 nm, as described in Materials and Methods. Data were expressed as ratio of the absorbance in empty vector-overexpressed ST2 cells. Data represent mean ± SEM (*n* = 8 in each group). **p < 0.01 by Dunnett test. Lower panel shows the representative images of mineralization

**Fig. 4:**
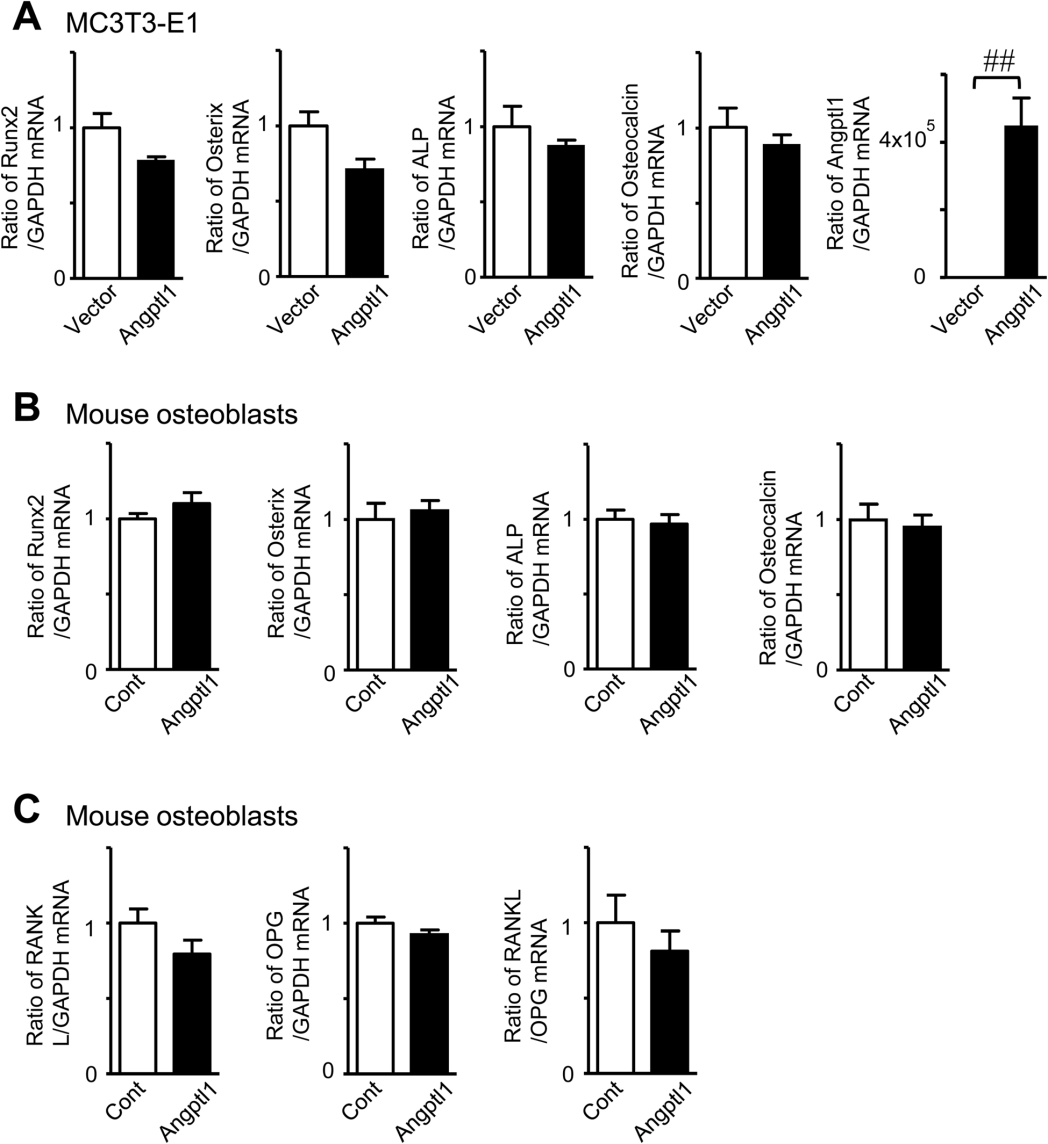
Effects of Angptl1 on osteoblast phenotype. **a** Total RNA was extracted from subconfluent MC3T3-E1 cells transiently transfected with empty vector or Angptl1. Then, qRT-PCR for Runx2, Osterix, ALP, osteocalcin, Angptl1 or GAPDH was performed. Data are expressed relative to the GAPDH mRNA value. Data represent mean ± SEM (*n* = 8 in each group). ^##^*p* < 0.01 by Mann-Whitney U test. **b** Mouse osteoblasts were cultured in the presence and absence of recombinant Angptl1 (100 ng/ml) for 24 h, and total RNA was extracted. Then, qRT-PCR for Runx2, Osterix, ALP, osteocalcin or GAPDH was performed. Data are expressed relative to the GAPDH mRNA value. Data represent mean ± SEM (*n* = 6 in each group). Cont; Control. (C) Mouse osteoblasts were cultured in the presence and absence of recombinant Angptl1 (100 ng/ml) for 24 h, and total RNA was extracted. Then, qRT-PCR for RANKL, OPG or GAPDH was performed. Data are expressed relative to the GAPDH mRNA value. Data represent mean ± SEM (*n* = 6 in each group). Cont; Control

We examined the effects of Angptl1 on osteoblastic differentiation using ST2 cells, mouse mesenchymal cell line. Transient overexpression of Angptl1 significantly enhanced Osterix mRNA levels and ALP activity enhanced by BMP-2 in ST2 cells, although it significantly reduced osteocalcin mRNA levels enhanced by BMP-2 (Fig. 3a and b). Next, we established ST2 cells stably expressing Angptl1 to examine the effects of Angptl1 on mineralization. As shown in Fig. 3c, stable overexpression of Angptl1 enhanced mineralization in ST2 cells pretreated with BMP-2 in the presence of mineralization medium. On the other hand, transient overexpression of Angptl1 did not affect osteoblast phenotypes, such as the mRNA levels of Runx2, Osterix, ALP and osteocalcin in mouse osteoblastic MC3T3-E1 cells (Fig. 4a). Recombinant Angptl1 protein did not affect the mRNA levels of Runx2, Osterix, ALP and osteocalcin in mouse osteoblasts (Fig. 4b). Moreover, Angptl1 did not affect the mRNA of RANKL and osteoprotegerin (OPG) as well as the ratio of RANKL/OPG mRNA in mouse osteoblasts (Fig. 4c).

### Effects of Angptl1 on adipogenic differentiation

**Fig. 5:**
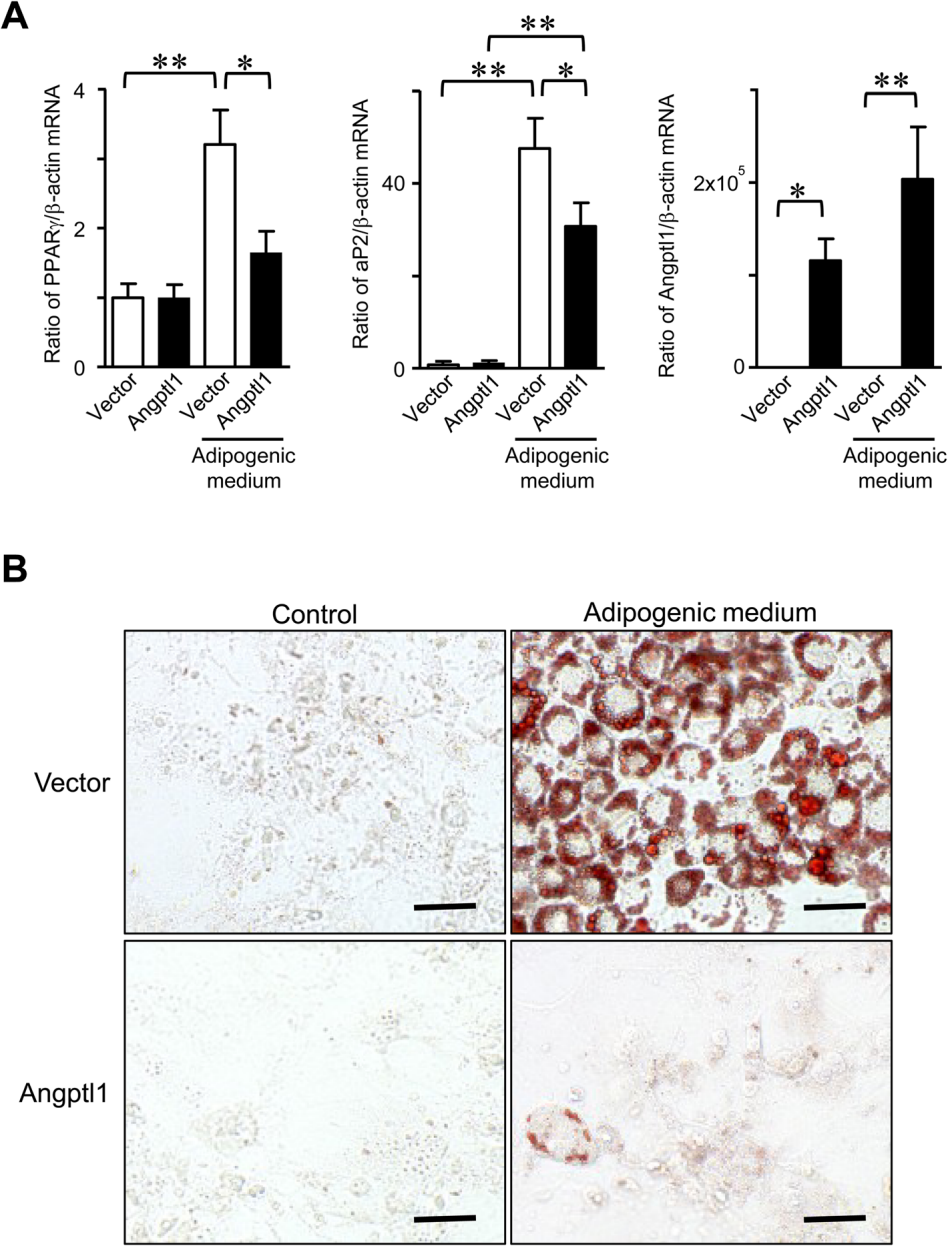
Effects of Angptl1 on adipogenic differentiation. **a** Total RNA was extracted from transiently empty vector- or Angptl1-transfected 3T3-L1 cells with and without adipogenic medium (10 μg/ml insulin, 500 μM 3-isobutyl-1-methyl-xanthine and 1 nM dexamethasone) for 6 days. Then, qRT-PCR for PPARγ, aP2, Angptl1 or β-actin was performed. Data are expressed relative to β-actin mRNA value. Data represent mean ± SEM (*n* = 6 in each group). *p < 0.05, **p < 0.01, by Tukey-Kramer test. **b** Transiently empty vector- or Angptl1-transfected 3T3-L1 cells with and without adipogenic medium for 6 days. Then, cells were stained with Oil red O. Data shows the representative images. Scale bar indicates 50 μm. At least three independent experiments were performed

We finally examined the effects of Angptl1 on adipogenic differentiation. Mouse preadipocyte cell line, 3T3-L1 cells, transiently overexpressing Angptl1, differentiate to adipocytes in the presence of adipogenic medium for 4 days. Transient overexpression of Angptl1 significantly decreased the mRNA levels of peroxisome proliferator-activated receptor γ (PPARγ) and adipocyte protein-2 (aP2) enhanced by adipogenic medium in 3T3-L1 cells (Fig. 5a) as well as Oil Red O staining (Fig. 5b).

## Discussion

The present study revealed that Angptl1 overexpression significantly inhibited number of TRAP-positive MNCs as well as the mRNA levels of TRAP and Ctsk enhanced by RANKL in RAW 264.7 cells. Moreover, Angptl1 protein significantly suppressed number of TRAP-positive MNCs as well as the mRNA levels of TRAP, Ctsk, NFATc1, MMP-9 and DC-STAMP enhanced by RANKL in mouse bone marrow cells. These findings indicate that Angptl1 suppresses osteoclast formation, then might lead to a suppression of bone resorption. Since the effects of Angptl1 on NFATc1 expression seemed to be less than on those of TRAP and Ctsk in RAW 264.7 cells, Angptl1 might differently affect several signaling for osteoclast formation in mice. It has previously been reported that Angptl1 exerts anti-apoptotic activity through the phosphorylation of ERK1/2 and Akt in zebrafish endothelial cells [11]. Moreover, the studies of Akt-deficient mice suggested that Akt signaling is partly related to osteoblastic bone formation and osteoclastic bone resorption in bone remodeling [17, 18], although the previous studies suggest that ERK1/2 and Akt signaling stimulates bone resorption in mice in contrast to the effects of Angptl1 on osteoclast formation [19]. The roles of Angptl1 in osteoclast function have remained unknown. Further studies, such as pit formation assay using bone slices, will be necessary to clarify the roles of Angptl1 in bone resorption activity.

Although MC3T3-E1cells are preosteoblastic cells, which already possess the phenotype with the osteoblastic character, such as high ALP activity and mineralization, ST2 cells are stromal cell-line, which do not exhibit the osteoblast phenotype. BMP-2 can induce the differentiation of ST2 cells into osteoblastic cells. In the present study, Angptl1 overexpression significantly enhanced Osterix mRNA levels, ALP activity and mineralization in the presence of BMP-2 in ST2 cells, although it did not affect the mRNA levels of Runx2, Osterix, ALP and osteocalcin in mouse osteoblasts and MC3T3-E1 cells. These data suggest that Angptl1 enhances the mesenchymal cells into osteoblasts at the early stage of osteogenic differentiation, then leading to an enhancement of ALP activity and mineralization. In the present study, Angptl1 overexpression significantly suppressed osteocalcin mRNA levels enhanced by BMP-2 in ST2 cells, suggesting that Angptl1 affects osteoblastic differentiation at the early stage, but not the terminal stage, since osteocalcin has been considered to be late stage osteoblast differentiation marker [20].

Angptl2 has been well-studied in glucose/lipid metabolism. Kadomatsu et al. demonstrated that Angptl2 possesses angiogenic effects, which are related to the chronic inflammation in obesity and cancer metastasis [21–23]. Serum levels of Angptl2 have also been reported in type 2 diabetic and obese patients [24, 25]. These in vitro and clinical studies suggested that Angptl2 is involved in the pathophysiology of metabolic syndrome, such as obesity, although no reports are available about the roles of Angptl1 in adipobiology. We therefore examined the effects of Angptl1 on adipogenic differentiation, since bone metabolism is regulated by the trans differentiation of mesenchymal stem cells into osteogenic or adipogenic cells [26]. In the present study, Angptl1 overexpression significantly decreased the mRNA levels of PPARγ and aP2 as well as the formation of lipid droplets during adipogenic induction in mouse preadipocyte cell line, 3T3-L1 cells, suggesting that Angptl1 suppresses adipogenic differentiation in mouse cells. Taken into account into the enhancement of Angptl1 on the mesenchymal cells into osteoblasts at the early stage, Angptl1 induces trans differentiation from adipogenic-lineage into osteogenic lineage cells during bone remodeling or in the pathological state, such as diabetes and obesity.

Bone modeling and remodeling process participate during bone repair after fractures or bone defect [27]. Chondrogenesis, bone formation and bone resorption as well as vessel formation and inflammation are related to this bone repair process. Tanoue et al. evaluated Angptl2 expression and function in chondrocyte differentiation and subsequent endochondral ossification in mice, and they showed that Angptl2 functions as an extracellular matrix protein in chondrocyte differentiation and subsequent endochondral ossification [28]. Therefore, Angptl1 might participate bone repair process by affecting chondrogenesis, bone restoration and remodeling phase, although our preliminary study showed that an elevation in Angptl1 mRNA level are not evident at the injured sites after femoral bone defect in mice (data not shown). The roles of Angptl1 in chondrogenesis and bone repair have still remained unknown.

## Conclusions

In conclusion, the present study first revealed that Angptl1 overexpression suppresses osteoclast formation enhanced by RANKL in RAW 264.7 cells and mouse bone marrow cells. Moreover, it enhanced Osterix expression, ALP activity and mineralization induced by BMP-2 in ST2 cells, although it suppressed adipogenic differentiation of 3T3-L1 cells. This is the first report about the roles of Angptl1 in bone. Our data suggest the possibility that Angptl1 might enhance and reduce osteoblastic bone formation and osteoclastic bone resorption in mice, respectively, which might leading to an increase in bone mass and a target of bone metabolic disorders, such as osteoporosis. Further in vivo study using Angptl1-deleted mice is required to clarify the roles of Angptl1 in bone metabolism in the future.

## Data Availability

The data sets used for the current study are available from the corresponding authors upon reasonable request.

## Abbreviations

aP2: Adipocyte protein-2
ALP: Alkaline phosphatase
αMEM: Minimum essential medium alpha modification
Angptl1: Angiopoietin like protein-1
Angptls: Angiopoietin like proteins
BMP-2: Bone morphogenetic protein-2
Ctsk: Cathepsin K
DC-STAMP: Dendrocyte expressed seven transmembrane protein
DMEM: Dulbecco’s modified eagle’s medium
ERK1/2: Extracellular-signal-regulated kinase 1/2
FBS: Fetal bovine serum
GAPDH: Glyceraldehyde-3-phosphate dehydrogenase
MMP: Matrix metalloproteinase
MNCs: Multinuclear cells
NFATc1: Nuclear factor of activated T-cells, cytoplasmic 1
PPARγ: Peroxisome proliferator-activated receptor γ
qRT-PCR: Quantitative reverse transcriptase-polymerase chain reaction
RANKL: Receptor activator of nuclear factor κB ligand
TRAP: Tartrate-resistant acid phosphatase
